# Hysteroscopy vs. Vabra in Endometrial Cancer Diagnosis: A Systematic Review of the Literature

**DOI:** 10.3390/cancers17071145

**Published:** 2025-03-28

**Authors:** Christopher Clark, Ambrogio Cazzolla, Giuseppe Colonna, Vera Loizzi, Gennaro Cormio, Salvatore Lopez

**Affiliations:** 1 Division of Obstetrics and Gynecology, Department of Biomedical and Human Oncological Science, University of Bari “Aldo Moro”, 70124 Bari, Italy; 2 S.C. Ginecologia Oncologica Clinicizzata, IRCCS Istituto Tumori “Giovanni Paolo II”, 70124 Bari, Italy; a.cazzolla@oncologico.bari.it (A.C.); g.colonna@oncologico.bari.it (G.C.); v.loizzi@oncologico.bari.it (V.L.); g.cormio@oncologico.bari.it (G.C.); salvatore.lopez@hotmail.it (S.L.); 3 Dipartimento di Biomedicina Traslazionale e Neuroscienze (DiBraiN), University of Bari “Aldo Moro”, 70124 Bari, Italy; 4 Dipartimento DIM, University of Bari “Aldo Moro”, 70124 Bari, Italy

**Keywords:** endometrial cancer, endometrial biopsy, VABRA, vacuum biopsy hysteroscopy

## Abstract

**Introduction:** Endometrial cancer is the most common malignancy of the female genital tract in high-income countries. A prompt diagnosis of this condition is of utmost importance in ensuring that patients receive the best treatment strategy. The new FIGO 2023 molecular classification of endometrial cancer radically changes the current landscape of this disease’s treatment and follow-up. Among the various diagnostic techniques used to identify endometrial lesions, hysteroscopy and vacuum biopsy techniques are the most employed in clinical practice. The aim of this systematic literature review is to compare the efficacy, sensitivity, specificity, and safety of these methods when employed to diagnose endometrial cancer, as well as to assess the feasibility of endometrial cancer molecular profiling on biopsy specimens. **Methods:** A systematic literature search of studies evaluating hysteroscopy and VABRA biopsy performance in diagnosing endometrial cancer was conducted using the main online databases (PubMed, EMBase, Cochrane Library). An additional literature search was conducted, focusing on the feasibility of endometrial cancer molecular profiling on hysteroscopic biopsy and VABRA samples, as well as on the diagnostic concordance of biopsy and final surgery specimens. Two authors performed the literature search independently, while other two authors assessed the retrieved publications’ risk of bias using Begg’s and Egger’s tests. Twenty-four studies were included in the final report. **Results:** Both techniques have shown high effectiveness in diagnosing endometrial cancer, although with important differences. Hysteroscopy provides direct visualization of the endometrium and allows clinicians to perform biopsies of suspicious lesions, but it also entails important limitations, as in the case of diffused lesions or technical difficulties, especially in nulliparous and elderly women. VABRA and other vacuum biopsy techniques, on the other hand, offer a wider sampling of endometrial tissue and are less operator-dependent but may be associated with a higher risk of failed diagnosis when compared to hysteroscopy. **Discussion:** Hysteroscopy, especially when combined with targeted biopsies, has been proven to be a valid technique, especially in pre-surgical diagnostic workup of early-stage endometrial cancer. Vacuum techniques, although less invasive and providing larger tissue samples, do not seem to be able to completely replace hysteroscopy in diagnosing endometrial cancer but remain a valid alternative in selected cases, especially when endometrial lesions prove harder to reach and/or to identify. They may also prove useful in low resource countries in which hysteroscopy is not widely available. **Conclusions:** Based on our findings, early assessment of endometrial cancer should be carried out through hysteroscopic evaluation and targeted endometrial biopsies, ideally including molecular assessment on biopsy specimens to further guide treatment decisions. Other biopsy techniques, such as vacuum-assisted biopsy, should be reserved for specific settings in which hysteroscopy is not readily available. The ideal diagnostic approach to endometrial cancer should take multiple factors into account, such as the location and extension of the disease, patient characteristics, clinical skills, and resource availability. Further studies are needed to assess the feasibility of molecular cancer profiling on biopsy specimens, as well as cost-containment strategies which would allow equal access to targeted treatment modalities for all endometrial cancer patients worldwide.

## Simple Summary

Endometrial biopsy is one of the most important steps in diagnosing endometrial cancer, for it allows clinicians to differentiate this disease from other possible causes of abnormal uterine bleeding and to obtain critical information on both histological and molecular features of endometrial cancer. Moreover, a failed biopsy procedure or an inadequate biopsy specimen may delay patient treatment. Many options are available for this purpose in clinical practice, although the most commonly used ones are hysteroscopy and the vacuum biopsy technique, such as Pipelle and VABRA. This review focuses on comparing the diagnostic accuracy, complication rate, and cost-effectiveness of these techniques, aiming to provide the most up-to-date information on the topic and allow clinicians to perform evidence-oriented choices when referring a patient to endometrial biopsy procedures.

## 1. Introduction

Endometrial cancer (EC) is one of the most common gynecological malignancies worldwide, with an estimated number of 67,880 new cases expected to be diagnosed by the end of 2024 in the U.S. alone [1]. Key risk factors in EC development lie in hormonal imbalances which determine a prolonged exposure to estrogen stimulation, such as nulliparity, obesity, and pre-existing medical conditions such as diabetes mellitus [2]. In high-income countries, endometrial cancer is the most common malignancy of the female genital tract.

The most important clinical features of EC are Abnormal Uterine Bleeding (AUB) or abnormal vaginal discharge in peri- or post-menopausal women, which often prompt gynecological assessment [3]. Trans-Vaginal Ultrasound (TVUS) examination is an important diagnostic tool used to examine these patients, as it provides information regarding endometrial thickness and shape, the status of the endo-myometrial junction, and endometrial blood supply through Doppler evaluation [4].

Upon recognizing ultrasonographic patterns of suspected endometrial malignancy, an invasive procedure is often required in order to obtain pathology specimens and confirm the diagnosis. Different techniques are available for this purpose, the most commonly used being office or inpatient hysteroscopy and Vacuum Aspiration Biopsy Random Assay (VABRA).

Hysteroscopy is a minimally invasive gynecological endoscopic procedure which consists of inserting a rigid or flexible probe inside the uterus through the vagina and the cervix [5]. The main advantage of hysteroscopic evaluation of the uterine cavity lies in the possibility of performing targeted biopsies, thus obtaining pathology specimens directly from suspicious areas. However, this procedure requires a long learning curve and extensive training, as it may be associated with numerous complications, especially when performed by inexperienced clinicians [6]. Moreover, while generally well-tolerated, hysteroscopy may prove too painful for certain subgroups of patients (especially elderly and nulliparous women) and hysteroscopic equipment may not be easily available.

VABRA, on the other hand, consists of a simple and easily reproducible procedure in which endometrial samples are obtained through a 3 mm diameter aspirator, inserted in the uterine cavity through the cervix. Endometrial tissue is then harvested from the uterine cavity using a suction technique [7]. The advantages of this method are its gentle learning curve, greater patient compliance, and contained costs, although these advantages are offset by a theoretical decrease in diagnostic accuracy.

Recent advances in molecular biology have shed new light on endometrial cancer, which have culminated in the new FIGO 2023 EC staging. The most important difference between the previous and the latest FIGO staging is the recognition of different molecular patterns of the disease, which indicate different cancer biology, treatment response and prognosis [8]. A priori knowledge of EC molecular subtypes could be useful not only in tailoring post-operative treatment and follow-up, but also in surgical management, by selecting cases which could benefit from sentinel lymph node biopsy. Concordance between final surgery histology and biopsy, however, is not clear [9].

The purpose of this paper is to review the available literature regarding different endometrial biopsy techniques so as to compare their efficacy, sensitivity, specificity, and safety in diagnosing endometrial cancer, as well as the feasibility of EC molecular profiling on biopsy specimens. Our scope is to determine whether EC diagnosis and subsequent molecular profiling could be achieved through different endometrial biopsy techniques, which may be more readily available in extra-hospital settings such as outpatient clinics and potentially prove to be cheaper and safer alternatives to hysteroscopy.

## 2. Materials and Methods

A comprehensive literature search was performed independently by four authors associated with this current study, in full compliance with the PRISMA guidelines, using the following electronic databases: PubMed, Embase, Medline, and the Cochrane Library. The last literature search was conducted on 13 January 2025. The language of studies was limited to English alone. Two authors (C.C., S.L.) performed the literature search independently, while two other authors (V.L., A.C.) assessed the quality of the retrieved publications by analyzing each study’s methods, endpoints, and results by using Begg’s and Egger’s tests to ensure a low risk of bias.

**Figure 1:**
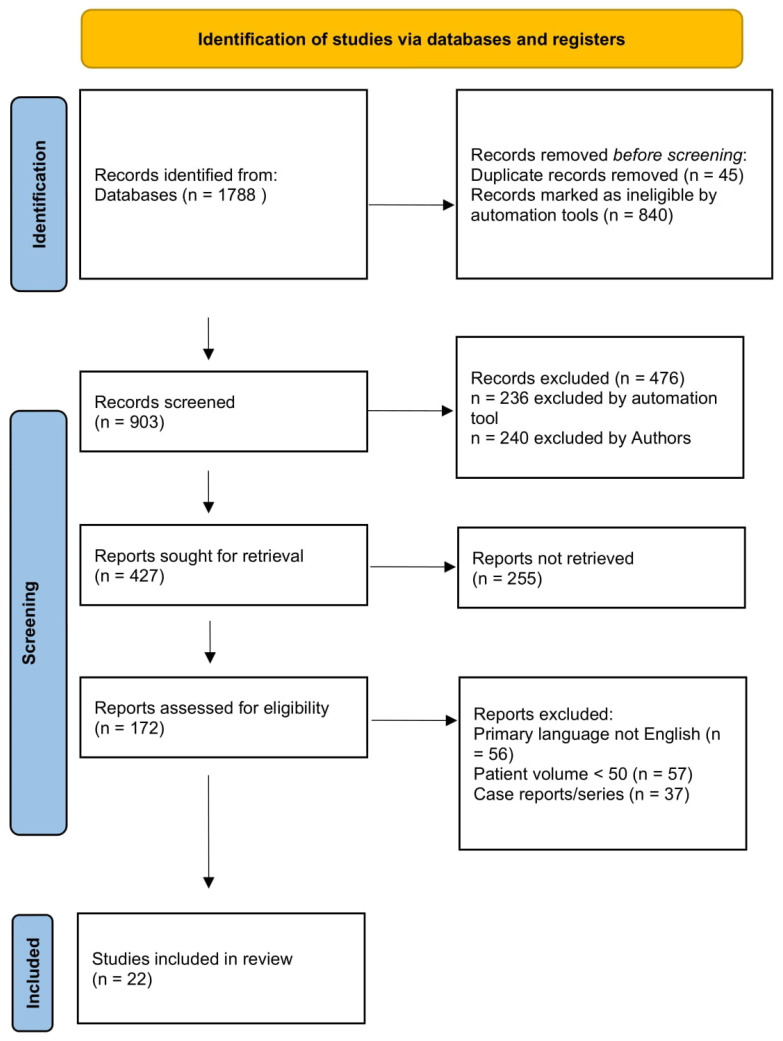
Preferred Reporting Items for Systematic reviews and Meta-Analyses (PRISMA) flow diagram of study selection.

The predefined keywords used for the search were “endometrial cancer”, “molecular”, “hysteroscopy”, and “VABRA”. To perform the literature search detailed in this study, a search algorithm was used to select and screen results based on a combination of the following search terms: “molecular AND (endometri* OR uterus OR uterine) AND (Hysteroscopy OR Biopsy* OR VABRA* OR Vacuum*)”. A further screening of retrieved publications was subsequently performed to include only relevant results. In this review we included prospective observational cohort studies, retrospective studies, and meta-analyses only. Exclusion criteria for this study were as follows: case reports, case series, and studies with low patient volume (<50 patients), to reduce the risk of bias and include only studies with high statistical power. A summary of the study methods is provided in Figure 1.

## 3. Results

The studies included in the present literature review, the number of participants, the main objective of each study, and their results are summarized in Table 1.

### 3.1. VABRA Aspirator Performance

Many aspiration devices are available for clinical employment in diagnosing endometrial lesions. One of the most used devices is the VABRA aspirator, which consists of a metal cannula measuring 24 mm in length and 3 mm in external diameter, which is inserted in the uterine cavity through the cervix. Endometrial tissue is harvested by connecting the cannula to an aspiration tube, which drains into a plastic receptacle.

Recent literature investigating the performance of the VABRA aspirator is scant. Nonetheless, the older studies, which can be found in the literature, are of great importance, since they remain the sole means of comparability between current gold standard biopsy techniques and alternative biopsy methods, as the clinical employment of vacuum devices has greatly decreased over the years. A 1982 paper by Einerth [10] explored clinical performance of the VABRA aspirator on 332 patients in which endometrial biopsy was indicated. A total of 296 patients were eligible for the procedure and sufficient material for histological examination was collected from 276 patients. Benign conditions were diagnosed in most cases, and endometrial cancer was present in only 7 patients (2.4%). During a 1.5–5-year follow-up, only 16 patients required subsequent diagnostic evaluation due to uterine bleeding, and endometrial cancer was not present in any of these patients. Regarding patient compliance and post-procedural complication, the author reported moderate and severe pain in 45 and 7 patients, respectively, and vaso-vagal reaction in 2 patients. Furthermore, one patient developed endometritis, while six patients reported uterine bleeding following the procedure, concluding that the VABRA aspirator is a safe and cost-effective procedure with satisfactory reliability.

Another paper by Lubbers [7] investigated the matter of diagnostic reliability of the VABRA aspirator. In this paper, 262 patients were divided into four different groups and underwent VABRA biopsy for different clinical indications, including post-menopausal bleeding and recurrent miscarriages. Each group referred to different clinical settings; for example, group A consisted of 124 patients who underwent VABRA biopsy first and conventional Dilation and Curette (D&C) biopsy the day after, while group B was made up of 81 patients pending hysterectomy who underwent VABRA biopsy the day before their scheduled surgery. These two groups were particularly important due to the possibility of comparing the results of biopsy specimens obtained by VABRA aspiration with conventional biopsy methods (group A) and final histology (group B). The results of this study showed that VABRA biopsy had excellent concordance with conventional biopsy techniques and with final histology samples, and most importantly no malignancy was missed by the VABRA technique.

Another study by Kaunitz et al. [11] compared sample adequacy of VABRA and Pipelle aspirators by recruiting 56 patients and performing both techniques on each patient. Both techniques rendered the correct diagnosis in 50 patients (89%), although the authors reported that Pipelle sampling caused less pain and thus improved patient compliance in 50 patients.

These findings, although encouraging, reflect an outdated landscape of endometrial biopsy techniques and do not allow any direct comparison with modern biopsy modalities. In fact, there are no studies in the literature which directly compare the results of VABRA versus hysteroscopic endometrial biopsies, nor are there updated studies which demonstrate a concordance between VABRA biopsy and final pathology specimens. No clear conclusion can be drawn from the available literature regarding the feasibility of VABRA biopsy without jeopardizing oncological outcomes.

### 3.2. Hysteroscopy Performance

As the name suggests, hysteroscopy is a technique which allows visualization of the uterine cavity, which is achieved by inserting a rigid endoscope inside the uterus through the cervical os. Uterine wall distension is achieved through saline solution, which is irrigated directly by the hysteroscope and allows visualization of both uterine walls, cervical canal, and tubal recesses. In addition, most hysteroscopes are equipped with working channels which allow the introduction of micro-forceps, thus allowing clinicians to perform targeted biopsies.

The diagnostic accuracy of hysteroscopic evaluation has been extensively measured. An interesting prospective study by Angioni et al. [12] evaluated diagnostic performance of hysteroscopy in comparison to Novak’s curette in 319 patients with AUB who underwent both procedures from January 1992 to October 2004. Patients then underwent final surgery depending on their specific indication and the concordance between hysteroscopic/blind biopsy and final histology was assessed. Result showed that hysteroscopy-guided biopsy yielded better sensitivity, specificity, and diagnostic accuracy as compared to Novak’s curette in diagnosing endocavitary benign lesions, with almost perfect concordance with final histology reports.

Other studies have also clarified the accuracy of hysteroscopy in diagnosing endometrial hyperplasia. A famous report by Loverro et al. [13] explored the role of hysteroscopy in diagnosing this condition by comparing visual aspects of the endometrium (which suggest underlying endometrial hyperplasia) to final histology reports. At the time in which the study was published, no hysteroscopic criteria had been established to diagnose endometrial hyperplasia, and therefore the reliability of this technique was still questioned. The authors collected data from 980 patients suffering from AUB which underwent hysteroscopic evaluation from 1993 to 1995 in the Obstetrics and Gynecology Department of Bari, Italy. All procedures were performed by the same gynecologist using the same office hysteroscope. Visual diagnosis of endometrial hyperplasia was formulated when one or more of the following criteria was present: focal or diffuse increase in the endometrial thickness, irregular aspect of the endometrial surface, button-like proliferations, or large protruding cyst in the uterine cavity, dilated glandular opening of yellowish color, large superficial vessels on the panoramic view. Focal areas suspected of underlying endometrial hyperplasia were biopsied and final histology reports were obtained. A comparison of hysteroscopic and histological diagnosis of endometrial hyperplasia was performed with McNemar test using histological diagnosis as the true result. A total of 128 patients out of 980 (13%) received hysteroscopic diagnosis of endometrial hyperplasia, and in 81 cases (63% Positive Predictive Value, PPV) histology reports confirmed the diagnosis. Overall, the authors reported hysteroscopy’s sensitivity and specificity in the diagnosis of endometrial hyperplasia to be 98% and 95%, respectively. Moreover, the authors reported a higher PPV (72% vs. 58%) of hysteroscopic diagnosis of endometrial hyperplasia in post-menopausal women, due to the absence of confounding hormonal-related endometrial aspects in fertile patients. Only two cases of endometrial hyperplasia were missed by hysteroscopy: in these two cases, hysteroscopic evaluation of the endometrium suggested endometrial carcinoma, and final hysterectomy reports was significant for well-differentiated endometrial adenocarcinoma with multiple areas of atypical hyperplasia. Based on these findings, the authors reported a Negative Predictive Value (NPV) of 99% for hysteroscopic diagnosis of endometrial hyperplasia, and concluded that the high accuracy of this technique, along with its minimal invasiveness, were ideal for both diagnosis and follow-up of conservative management of endometrial hyperplasia.

The role of hysteroscopic evaluation in diagnosing endometrial cancer has also been investigated by different studies. A study by Zhu et al. [14] retrospectively compared the performance of hysteroscopy in diagnosing endometrial cancer as compared to traditional Dilation and Curettage (D&C). To this purpose, data from 287 patients treated for endometrial carcinoma from 2002 to 2010 were obtained. Patients were divided into two groups: group A included 90 patients who underwent preoperative hysteroscopy, while group B included the remaining 197 patients who underwent fractioned D&C before surgery. Data from these patients showed that hysteroscopic biopsy successfully diagnosed endometrial cancer in 88 out of 90 patients, yielding a diagnostic accuracy of 97.8%, while D&C diagnosed 175 out of 197 patients with endometrial cancer. This study has drawn strong criticism for the difference in size of the two patient groups, with group B consisting of more than double the population of group A. When comparing the two methods’ ability for detecting cervical involvement in patients with endometrial cancer, however, results showed much higher sensibility, specificity, negative predictive value, and positive predictive value in the hysteroscopy arm. Another interesting finding of this study was the fact that, despite the use of saline solution to distend the uterine cavity (with consequent theoretical risk of peritoneal spread of endometrial cancer through the Fallopian tubes), there was no statistical difference between the two methods in positive peritoneal cytology, thus concluding that hysteroscopy does not increase the risk of peritoneal spread of endometrial cancer.

A recent meta-analysis by Gkrozou et al. [15] collected data from 17 studies regarding hysteroscopy performance in diagnosing the four most common uterine abnormalities in patients with AUB: endometrial cancer, atypical hyperplasia, endometrial polyps, and submucous myomas. As regards endometrial cancer diagnosis, the authors found hysteroscopy to yield sensibility of 82.6% and specificity of 99.7%. This indicates that, despite previous reports which demonstrated higher sensitivity rather than specificity, hysteroscopy performs better in excluding this disease rather than confirming its presence. As acknowledged by the authors, this may be explained by the fact that the studies included in the meta-analysis were conducted on patients with endometrial thickness greater than 5 mm, which renders the patient population more specific.

Based on these findings, endometrial biopsy through hysteroscopy is nowadays considered the gold standard for the diagnosis of endometrial cancer as a cause of uterine bleeding in post-menopausal patients.

### 3.3. Complications

Different methods of endometrial biopsy entail different kinds of complications. Theoretically, direct visualization of the uterine cavity allows physicians to perform higher quality biopsies and avoid major complications such as uterine perforation and bleeding, as compared to blind endometrial biopsy. This advantage, however, is offset by other possible issues, such as intravasation syndrome.

A recent report by Elahmedawy et al. [30] explored the different complications which can arise during or after hysteroscopy procedures. In this paper, the authors analyzed data from 71,000 patients who underwent hysteroscopy from 2019 to 2020 in England, almost half of which were therapeutic. Complications were divided into two groups, immediate/early- and late-onset, depending on the moment in which they arose. The most frequent immediate and early complications were uterine bleeding, uterine perforation, and cervical laceration, which are shared with blind biopsy methods. Although common, these complications are more frequent in the operative setting and when the procedure is performed by inexperienced surgeons. Other frequent early-onset complications are arrhythmias (which are due to cervical dilation and therefore also more common in the operative setting) and fluid overload-related syndromes such as hyponatremia and glycine toxicity. These complications are specifically related to hysteroscopy and depend on operating time and distention media. In outpatient and office hysteroscopy settings, saline solution is the preferred distention medium, and operating time is generally limited to 5–10 min, therefore the incidence of said complications is low.

As for long-term complications of office hysteroscopy, a retrospective cohort study by van Keervorde et al. [16] analyzed data from 1028 patients who underwent office hysteroscopy from January 2005 to October 2007. In total, 622 procedures (60%) were diagnostic, 328 (32%) were therapeutic, and 78 (8%) procedures failed. In 72 cases complications after hysteroscopy were reported; however, none of these were considered long-term complications. Only one case of office hysteroscopy was admitted 9 days after the procedure for suspicion of a complication, although subsequent inpatient diagnostic workup was inconclusive. Based on this study’s findings, the authors reported a long-term complication risk of 0.001% (1/1028) over a follow-up period of 1 year, once again demonstrating the safety of office hysteroscopic procedures.

Another theoretical complication of diagnostic hysteroscopy is spreading cancer in patients with endometrial cancer. The main concern surrounding this possibility is the fact that distension media may cause cancerous cells to spread into the abdomen through the fallopian tubes.

The safety of hysteroscopic evaluation of endometrial cancer has been demonstrated by many studies. In 2003, Selvaggi et al. [17] published a retrospective chart review in which data from 147 patients who underwent surgery for endometrial carcinoma from years 1995 to 2000 were collected. Almost all patients underwent hysteroscopy before surgery to either diagnose endometrial cancer or study cervical involvement in order to guide subsequent surgical approach; in 52 patients, however, hysteroscopy was not performed due to organizational issues, although these patients had already received histological diagnosis of endometrial cancer through D&C biopsy and later referred to the authors’ institution for surgical treatment. Patients were subsequently divided into three groups: D&C alone (n = 52, 35%), D&C plus hysteroscopy (n = 56, 39%), and hysteroscopy alone (n = 39, 26%). Patient distribution was casual since most patients who received endometrial cancer diagnosis through D&C were diagnosed elsewhere and later referred to the authors. 

After surgical treatment, extrauterine spread of EC was found in 30 patients: 9 of these had positive peritoneal cytology, 13 had microscopic ovarian metastases, and 8 showed microscopic peritoneal and omental metastases. No statistically significant differences in distribution of extrauterine disease in relation to the different diagnostic techniques were reported by the authors, suggesting that hysteroscopy does not increase the risk of EC spread when compared to D&C.

A systematic review and meta-analysis by Du et al. [18] recently investigated the topic of oncological safety of office hysteroscopy. The results of this study demonstrated that, although office hysteroscopy may in fact cause intraperitoneal spread of cancer cells, this does not significantly affect patient DFS or OS in early-stage EC. However, the low patient volume, the retrospective nature of the studies included in the meta-analysis, and the possible interference of adjuvant treatments may have influenced the results. This evidence prompts the need for further studies investigating the oncologic safety of office hysteroscopy and other biopsy techniques.

Up-to-date information regarding the rate of complications in VABRA biopsy is scarce. The most recent study investigating this topic dates back to 1980 and was performed by Delke et al. [19]. In this paper, the authors described the results of office VABRA aspiration which was performed in 271 patients over a 4-year period (1976–1979), with focus on indications, biopsy results, and reported complications. The most common issue reported by the patients was pain, which was identified as mild by 174 (64.2%), moderate by 74 (27.3%), and severe by 13 (4.8%) patients. Other complications such as infection and suspected uterine perforation were much less common. Syncope immediately after the procedure was reported in two cases, although both recovered within 5 min. The most important drawback regarding VABRA aspiration is the rate of insufficient tissue for histopathological examination, which was the fifth most common final diagnosis on endometrial samples collected through VABRA aspiration (13 cases, 4.8%) as well as the fact that only 50% of endometrial polyps were correctly diagnosed by this method in this case series, suggesting a lower diagnostic performance as compared to other sampling methods as already discussed.

### 3.4. Cost-Effectiveness

As reported by Warring et al. [31], diagnostic work-up in patients with AUB harbors great costs for healthcare systems, with higher costs deriving from instruments with sterilization costs such as hysteroscopy equipment and operating rooms, thus prompting the need for alternative diagnostic tools without jeopardizing diagnostic accuracy.

As mentioned above, VABRA aspiration biopsy is a simple procedure which requires very limited equipment consisting of an aspiration cannula and a mechanically or electrically operated vacuum device. By contrast, hysteroscopy equipment is much more complex as it requires an irrigation device, a light source, a camera, video support, and hysteroscopic instruments. Therefore, the cost of VABRA biopsy in outpatient setting seems, at least theoretically, lower than that of office hysteroscopy.

A study by Naim et al. [20] investigated cost-effectiveness of VABRA biopsy in 147 patients who underwent VABRA vs. Pipelle outpatient biopsy. The authors found that, although the price of a single VABRA kit was two times lower than the price of a single Pipelle cannula, the average procedure cost per patient was higher in the VABRA group due to procedure failure. This could be explained by the fact that the Authors used a 2.5 mm caliber aspiration cannula which was provided with the VABRA biopsy kit, which reduced patient discomfort but yielded less tissue volume for histological evaluation, thus requiring a second outpatient procedure with hysteroscopy. Moreover, the Pipelle device used in the aforementioned study was equipped with a piston rod inserted within the sheath which made the cannula stiffer and therefore more able to overcome small resistances when inserted into the cervix.

Another paper by Moawad et al. [21] investigated the cost-effectiveness of performing office hysteroscopy for abnormal uterine bleeding in 130 women and compared the costs of office and OR hysteroscopy. The results of this study showed that accurate patient selection reduced the need for OR hysteroscopy in 75 cases resulting in a significant reduction in costs per patient. One flaw of this study was that it specifically included only pre-menopausal women in which endometrial cancer is less likely to occur and in which office hysteroscopy is usually better tolerated due to anatomical and physiological differences in the cervical and endometrial tissue.

Cost-minimization analysis performed by Breijer et al. [22] investigated the possibility of creating a decision model which would select patients for direct endometrial biopsy or hysteroscopy-guided biopsy. In this study, two different strategies for PMB were analyzed—the first strategy consisted of performing endometrial biopsy in an outpatient setting, reserving office hysteroscopy for cases of failed biopsy, while the second strategy consisted of the creation of an algorithm which could predict endometrial biopsy failure based on patient characteristics, thus guiding clinicians in deciding whether to perform direct hysteroscopy or direct endometrial biopsy. Results of this study showed that no significant difference could be observed between the two strategies, meaning that deciding to perform hysteroscopy or direct endometrial biopsy based on patient characteristics does not improve cost-effectiveness. The authors also concluded that endometrial biopsy (through Dilation and Curettage, endometrial aspiration, or Novak biopsy) should be offered to any post-menopausal patient with AUB with US endometrial thickness of more than 4.0 mm, reserving office or OR hysteroscopy for failed biopsy cases.

As the available literature suggests, there is no standard approach which offers both diagnostic accuracy and cost-effectiveness in diagnosing endometrial cancer, and diagnostic strategies may vary depending on each country’s healthcare system and patient demographics. The most cost-effective strategy for diagnosing causes of AUB or PMB appears to be direct endometrial biopsy through D&C, Novak biopsy, or VABRA/Pipelle endometrial sampling, which offer satisfactory diagnostic accuracy. One major issue related to these methods is that blind biopsy of the uterine cavity does not allow direct visualization of the endometrium and thus reduces the chance of recognizing small lesions which could underline aggressive malignancies. Furthermore, issues related to low-volume samples may complicate the widespread use of VABRA aspiration techniques and result in misdiagnosis, which in turn prompts a second assessment through hysteroscopy and thus increases healthcare costs while reducing patient comfort and compliance. When prediction algorithms are used to select patients which could benefit from direct endometrial biopsy versus hysteroscopy-guided biopsy, no differences in cost-effectiveness are found. More research is needed to improve patient compliance in office hysteroscopy and better planning diagnostic modalities.

### 3.5. EC Molecular Profiling on Biopsy Specimens

EC is a polymorphous disease. Many molecular pathways can be involved in its pathogenesis; however, four major mutational patterns are found in most cases of EC [32]. Different mutational patterns not only imply different cancer biology but also significantly impact on patient prognosis and therapeutic strategies. 

As already discussed in a previous literature review [33], a priori knowledge of EC mutational status could benefit surgical treatment planning. In fact, some evidence suggests that Sentinel Lymph Node assessment during radical surgery could be safely avoided in some cases of EC with POLEmut molecular profile, although these data are not supported by prospective studies and conclusions were drawn on final pathology specimens.

Attempts to measure diagnostic concordance between endometrial biopsy and final pathology specimens in detecting EC molecular patterns began way before the introduction of the new FIGO 2023 molecular classification. Abdulfatah et al. [9] retrospectively analyzed data from 50 patients in which both preoperative and final pathology specimens were available. Each available H&E-stained specimen (both diagnostic and final) was reviewed by two independent gynecological pathologists to confirm the diagnosis. Furthermore, additional full sections from each specimen were obtained to perform ImmunoHistoChemical (IHC) analysis of mismatch repair (MMR) proteins and p53 status, as well as Sanger sequencing for polymerase epsilon (POLE). The Authors reported a high level of concordance for p53 and MMR proteins status (kappa 1.0), but POLE mutations were only detected in resection specimens. This study suggested the feasibility of pre-operative EC molecular profiling with the aim of better tailoring subsequent patient management. The lack of data regarding POLE mutated patients, however, did not allow the authors to draw any conclusions regarding pre-surgical management of these cases.

A recent article by Kato et al. [23] represented a new attempt to demonstrate concordance between molecular data harvested from histological and final pathology samples in patients with EC. The authors collected data from 70 patients who were treated for EC from 2012 to 2023. Furthermore, a special “POLE-mut enriched” cohort of patients was selected for analysis in order to obtain more information regarding POLE concordance between endometrial biopsy and final pathology. The results of this study showed a perfect concordance between pre-operative biopsy and final surgery in EC molecular profiling, although histological concordance rates between endometrial biopsy and final pathology were at best “moderate” (k = 0.420), in accordance with previous studies. Certainly, selection bias and the retrospective nature of this paper could explain the high concordance rates found by the authors; furthermore, POLE mutations were identified by analyzing all POLE exons: this method surely increases diagnostic accuracy but is matched by a sharp increase in costs and analysis time. Nonetheless, to the best of our knowledge, this study is the first in the literature to solve the low concordance rate for POLE status, as well as the first study to use whole-exome sequencing for POLE mutation assessment. As the authors themselves suggest, the use of POLE hotspot testing, which analyzes the most common mutations found in the Exonuclease Domain of Polymerase epsilon such as QPOLE [24], may overcome the limitations due to DNA sequencing.

In the age of precision medicine, EC molecular profiling remains one of the most outstanding examples of how the molecular characteristics of any type of oncological disease can be extremely helpful in tailoring the best treatment and follow-up approach for the affected patient. Although groundbreaking, the new FIGO 2023 molecular classification of endometrial cancer does not take into account the fact that EC molecular assessment is not carried out routinely in most areas of the world, as it would require specific equipment and a considerable economical investment; moreover, the role of pre-surgical molecular profiling in defining surgical treatment strategies for EC is poorly investigated in the literature. For all these reasons, we believe that further studies, preferably a comparison between oncological outcomes of patients who underwent molecular profiling before surgery for EC and those of patients who received standard treatment, are needed to guide future clinical practice.

## 4. Discussion

Endometrial sampling is a crucial step in assessing patients with AUB. To this purpose, various techniques have been proposed over the years, although hysteroscopy has been proven to be the most accurate. The most evident advantage of hysteroscopy is the possibility to perform targeted biopsies, which allow clinicians to obtain the most relevant information regarding areas of the endometrium which appear visibly suspicious. Moreover, this technique has proven to be safe and tolerated by most patients, although instrument-related costs may limit the use of hysteroscopy in low-income settings.

Vacuum aspiration is another minimally invasive technique which can provide useful information in patients with perimenopausal bleeding. The most used endometrial sampling methods of this kind are VABRA aspiration and Pipelle de Cornier. Although there are no recent studies which investigate these techniques’ accuracy in detecting endometrial diseases such as atypical hyperplasia or endometrial cancer, the available literature seems to suggest comparable results between vacuum aspiration techniques and more invasive procedures such as Dilation and Curettage (D&C). Complication rates correlated to the employment of these methods are low, and Pipelle devices seem to harvest more tissue and cause less patient discomfort when compared to other vacuum devices, although the scarce use of vacuum endometrial sampling in recent years has hampered data collection on the matter. Nonetheless, the data which can be retrieved from the available literature is of sound statistical significance and currently remains the sole means of comparison with modern biopsy techniques. Moreover, it is important to highlight that outpatient endometrial biopsy through vacuum aspiration gained great popularity in the past due to these technique’s high patient compliance and the relatively low need for clinician’s expertise in performing the procedure and the avoidance of general anesthesia. We hope that this review may be useful in sparking newfound interest in vacuum aspiration biopsy techniques and thus to better ascertain the current role of these methods in managing patients with abnormal uterine bleeding and endometrial cancer.

Sardo et al. [25] recently published a systematic review and meta-analysis in which blind endometrial biopsy and targeted endometrial biopsy through hysteroscopy were compared in terms of diagnostic accuracy. The aim of the study was to compare the ability of these different techniques’ sample adequacy and failure rates for the diagnosis of endometrial hyperplasia and cancer. After preliminary screening, four studies were included in the final dataset. The results of this study showed comparable rates of diagnostic accuracy between blind and targeted biopsy, although hysteroscopic endometrial biopsy is associated with significantly higher rates of sample adequacy. Despite these findings, many international societies still recommend the use of D&C or even Pipelle for obtaining histological diagnosis of endometrial cancer, and as acknowledged by the authors, the Society of Gynecologic Oncology and the American Congress of Obstetricians and Gynecologists still emphasize the diagnostic and therapeutic role of D&C [34,35].

Table 2 summarizes overall sensitivity, specificity, positive Likelihood Ratio [(+) LR], and negative Likelihood Ratio [(−) LR] of hysteroscopy and blind biopsy techniques in diagnosing endometrial cancer. As can be seen from the data shown below, hysteroscopy is highly accurate in diagnosing EC in patients with AUB; however, as noted by hysteroscopy’s high positive likelihood ratio, this technique’s high accuracy relates more to its ability to diagnose EC rather than excluding it. These findings are in contrast with those of other studies [15] which have shown the exact opposite, although selection bias may explain these results. Other techniques such as D&C and Pipelle yield a comparable specificity to that of hysteroscopy in diagnosing EC but worse (−) LR (and even lower sensitivity in the case of Pipelle biopsy). No conclusive data regarding the likelihood ratio of VABRA biopsy in endometrial cancer was found in the literature.

Cost analyses show that, although the price of the single aspiration technique is lower when compared to the costs of instrument sterilization, the indirect costs of biopsy failure due to blind biopsy techniques may offset this advantage. There are no currently accepted strategies to better manage cost-effectiveness, although, as some studies suggest, blind biopsy through aspiration techniques as an outpatient procedure may be used in resource-limited environments and selected cases to reduce costs of invasive procedures [29].

Other devices have been used to obtain endometrial samples both for suspected malignant and benign diseases. A recent literature review by Du et al. [36] explored different devices used for early assessment of suspected endometrial lesions. Apart from the more commonly used devices (D&C and vacuum devices), the review included data from other instruments such as Tao-brush and SAP-1 brush sampler.

Tao-brush was introduced in clinical practice in 1993 and consists of a thin plastic device equipped with a thin brush on the tip and is covered by an outer sheath. The outer sheath is pulled back and the tip of the instrument is inserted through the cervical os all the way to the fundus uteri; the instrument is then rotated of 360° 3–5 times to allow collection of endometrial cells. The outer sheath is subsequently pushed towards the tip and the inner brush is removed from the uterine cavity. Data analysis of the Tao-brush performance has shown specimen satisfaction ranging from 89.9% to 100%, pathological accuracy of 91–96%, and most importantly less specimen insufficiency when compared to Pipelle (2% vs. 12%); however, due to the brushes’ tip conformation, one limit of this device is its inability to collect specimens from the uterine horns.

The SAP-1 device, on the other hand, could theoretically solve this limitation due to the V-shape of its tip. The authors reported satisfactory diagnostic accuracy for this instrument, although there had not been enough clinical trials at the time the review was published to recommend extensive clinical employment of SAP-1. A summary of the authors’ findings regarding these endometrial sampling methods are found in Table 3.

Based on the findings of our study, early assessment of endometrial cancer should be carried out through hysteroscopic evaluation and aimed endometrial biopsies, ideally including molecular assessment on biopsy specimen to further guide treatment decisions. Other biopsy techniques such as vacuum-assisted biopsy should be reserved for specific settings, such as in the absence of hysteroscopic equipment and/or of trained endoscopic surgeons, or even in selected cases in which the patient does not tolerate hysteroscopy or when histological diagnosis is required but surgery is not considered as a treatment option due to patient comorbidities.

The landscape of endometrial cancer treatment is currently facing an unprecedented revolution. The importance of molecular assessment is now essential not only in managing post-surgical treatment and follow-up programs, but also in defining surgical strategies; therefore, a priori knowledge of EC molecular profile has become an impelling necessity. Recent literature suggests that the feasibility of EC molecular profiling on biopsy specimens, and the introduction of POLE hotspot testing kits may help reduce the cost of molecular testing, thus making POLE status assessment more accessible worldwide. Unfortunately, there are no studies in the literature which explore the feasibility of EC molecular testing on vacuum biopsy specimens, which could increase the chances of diagnosing EC and correctly manage this polymorphous disease even more.

## 5. Conclusions

This review summarizes the current evidence on the role of hysteroscopy and other blind endometrial sampling techniques, particularly vacuum-aspiration biopsy, in diagnosing endometrial cancer. The main limitations of this study include the limited availability of recent literature on vacuum device employment and the relatively small patient samples in the included studies. To the best of our knowledge, however, this study is the first to directly compare the performance of these techniques in terms of diagnostic accuracy, safety, and patient tolerability. Moreover, this study incorporates recent evidence on the feasibility of EC molecular profiling using biopsy specimens. The results of this review show that hysteroscopy offers a wide range of advantages when compared to blind biopsy techniques, such as the possibility of performing targeted biopsies, low complication rates, the possibility of performing the exam in outpatient settings, and high concordance rates between molecular analyses performed on hysteroscopically obtained endometrial biopsies and surgical specimens. The main limitations of hysteroscopy remain its steep learning curve and high costs, which could justify the employment of vacuum techniques in resource-limited environments. Nevertheless, this review once more highlights the great advantages of office hysteroscopy over other biopsy techniques in diagnosing endometrial cancer, in line with other studies which can be found in the literature. Although surgeon training is essential to achieve high diagnostic accuracy and patient compliance, hysteroscopy remains the most useful tool for assessing not only endometrial cancer but also other endometrial lesions such as polyps, fibroids, and endometrial adhesions. Furthermore, the introduction of micro-instruments and finer-caliber hysteroscopes allows to improve diagnostic accuracy even more and expands the possibilities of this procedure, even when performed with therapeutic intent.

## Figures and Tables

**Table 1:** Summary of studies included in the review.

Author, Year	Patients	Study Type	Main Objective	Results
Einerth et al., 1982 [10]	332	Prospective	VABRA performance on diagnosing endometrial lesions	VABRA biopsy has satisfactory reliability in diagnosing endometrial lesions
Lubbers et al., 1977 [7]	262	Prospective cohort study	To answer the question whether the procedure of the VABRA aspiration curettage without anesthesia is well tolerated by the patients; To assess the reliability of the VABRA curettage and to compare it with the conventional curettage in the diagnosis of various endometrial disorders.	VABRA is well tolerated and its reliability is high
Kaunitz et al., 1988 [11]	56	prospective	To compare VABRA and Pipelle performance in diagnosing endometrial cancer	Pipelle is comparable to VABRA in diagnosing EC
Angioni et al., 2008 [12]	319	Prospective trial without randomization	To compare blind endometrial biopsy (NOVAK) and direct visualization biopsy through hysteroscopy	Blind biopsy demonstrates low sensitivity and accuracy in benign focal intracavitary lesions. Hysteroscopy is confirmed as the gold standard in the assessment of abnormal uterine bleeding in menopause.
Loverro et al., 1996 [13]	980	Prospective without randomization	To determine the diagnostic accuracy of hysteroscopy in the diagnosis of endometrial hyperplasia in women with abnormal uterine bleeding	No case of endometrial hyperplasia was missed by hysteroscopy
Zhu et al., 2010 [14]	287	Retrospective	To compare hysteroscopy and D&C in the diagnosis of endometrial carcinoma	Hysteroscopy and directed biopsy offered improved pathological diagnostic accuracy before surgery and discovered cervical involvement more precisely in endometrial carcinoma patients, but it did not increase the positive peritoneal cytology rate or affect the prognosis of these patients
Gkrozou et al., 2014 [15]	9460	Meta-analysis	To determine the accuracy of hysteroscopy in diagnosing endometrial cancer, hyperplasia, polyps and submucous myomas	Hysteroscopy performs better in excluding endometrial cancer rather than confirming its presence
Van Kerkvoorde et al., 2012 [16]	1028	Retrospective cohort study	To estimate the long-term complication rate of office hysteroscopy with the vaginoscopic approach	Office hysteroscopy with the vaginoscopic approach is a safe procedure with an extremely low risk of long-term complications
Selvaggi et al., 2003 [17]	147	Retrospective	To determine the risk of peritoneal cancer spread after hysteroscopy	Hysteroscopy does not increase the risk of EC spread when compared to D&C
Du et al., 2021 [18]	3980	Meta-analysis	To explore the oncological safety of hysteroscopy for early-stage endometrial cancer	Hysteroscopy is a safe diagnostic and treatment method, and has no significant effect on the prognosis of early-stage endometrial cancer
Delke et al., 1980 [19]	320	Prospective	To evaluate endocervical curettage, uterine sounding, and vacuum aspiration in managing patients with post-menopausal bleeding, periodic follow-up of patients on long term estrogen therapy for postmenopausal symptoms and clinical staging and grading of endometrial cancer	VABRA complication rates are low, but its diagnostic accuracy is inferior when compared to other methods
Naim et al., 2007 [20]	147	Randomized prospective trial	To compare the effectiveness of the VABRA aspirator and the Pipelle device as an outpatient endometrial assessment tool	VABRA aspirator was not as effective as the Pipelle device in obtaining endometrial tissue for histological diagnosis. Despite its higher price per unit, the Pipelle device was a more cost-effective tool for outpatient endometrial assessment
Moawad et al., 2014 [21]	130	Retrospective cost-effectiveness study	To determine whether office hysteroscopy performed to evaluate abnormal uterine bleeding decreases the need for hysteroscopy performed in the OR and the associated financial and risk implications	Office hysteroscopy is a useful diagnostic tool that can help decrease the rate of diagnostic hysteroscopy in the OR under anesthesia when used in a select patient population
Breijer et al., 2015 [22]	356	Model based cost-minimization analysis	To evaluate whether a model to predict a failed endometrial biopsy in women with postmenopausal bleeding (PMB) and a thickened endometrium can reduce costs without compromising diagnostic accuracy	Individualizing the decision to perform an endometrial biopsy or immediate hysteroscopy in women presenting with postmenopausal bleeding based on patient characteristics does not increase the efficiency of the diagnostic work-up
Abdulfatah et al., 2019 [9]	60	Retrospective	To determine the level of concordance between endometrial biopsies and subsequent hysterectomy specimens in assessing the molecular classification of endometrial carcinoma.	A high level of concordance was achieved between biopsy and hysterectomy specimens for MMR-loss, MSI-high, P53-wild and abnormal types. Similar concordance could not be achieved for POLE mutation
Kato et al., 2024 [23]	70	Retrospective	to assess the concordance of molecular classifications between preoperative biopsy and hysterectomy to predict prognosis before surgical staging	Molecular subtypes were completely consistent with those derived from surgical specimens, demonstrating high concordance between preoperative and postoperative molecular classifications
Van den Heerik et al., 2023 [24]	284	Retrospective	To develop a quick, simple and reliable alternative for DNA sequencing and POLE mutation detection	QPOLE is a qPCR assay that is a quick, simple, and reliable alternative for DNA sequencing. QPOLE detects all pathogenic variants in the exonuclease domain of the POLE gene. QPOLE will make low-cost POLE-testing available for all women with EC around the globe
Di Spiezio Sardo et al., 2022 [25]	1295	Systematic review and meta-analysis	To compare endometrial biopsy performed under direct hysteroscopic visualization versus blind sampling for the diagnosis of endometrial hyperplasia and cancer	Hysteroscopic endometrial biopsy under direct visualization is associated with significantly higher rate of sample adequacy and is comparable to blind endometrial sampling for the diagnosis of endometrial cancer and precancer
Clark et al., 2002 [26]	26,346	Systematic review and meta-analysis	To determine the accuracy of hysteroscopy in diagnosing endometrial cancer and hyperplasia in women with abnormal uterine bleeding	The diagnostic accuracy of hysteroscopy is high for endometrial cancer, but only moderate for endometrial disease (cancer or hyperplasia)
Sakna et al., 2023 [27]	1607	Systematic review and meta-analysis	To determine the diagnostic accuracy of different endometrial sampling tests for detecting endometrial carcinoma.	High certainty evidence indicates that endometrial sampling using Pipelle or conventional D&C is accurate in diagnosing endometrial cancer. Studies assessing other endometrial sampling tests were sparse
Dijkhuizen et al., 2000 [28]	7914	Systematic review and meta-analysis	To assess the accuracy of endometrial sampling devices in the detection of endometrial carcinoma and atypical hyperplasia.	Endometrial biopsy with the Pipelle is superior to other endometrial techniques in the detection of endometrial carcinoma and atypical hyperplasia. The accuracy of the Pipelle is higher in postmenopausal women compared with premenopausal women
Tanko et al., 2021 [29]	180	Prospective cross-sectional study	To evaluate factors influencing the reliability and success rate of Pipelle endometrial sampling for histopathological diagnosis	Pipelle biopsy is a cheap, simple to handle, save, well tolerated, and a reliable office or outpatient tool. The more expensive procedures in the operating room should be reserved for selected patients who are not good candidates for Pipelle

**Table 2:** Diagnostic accuracy of different biopsy techniques in diagnosing endometrial cancer.

Biopsy Technique	Sensitivity	Specificity	(+) LR	(−) LR
Hysteroscopy	86.4% ^†^	99.2% ^†^	60.9 ^†^	0.15 ^†^
D&C	88% *	98.4% *	59.3 *	0.194 *
VABRA	66.7% ^‡^	99.8% ^‡^	N/A	N/A
Pipelle	77.4% *	98.5% *	97 *	0.247 *

Note: ^†^ adapted from [26]; * adapted from [27]; ^‡^ adapted from [28]. Each value is expressed as the percentage of concordance between histological samples obtained through endometrial biopsy and final histology obtained on gross specimens.

**Table 3:** Alternative devices for early assessment of suspected endometrial lesions.

Instrument	Sensitivity	Specificity	Positive Predictive Value (PPV)	Negative Predictive Value (NPV)
Tao-brush	95.5% *	100% *	100% *	98% *
SAP-1	73% ^†^	95.8% ^†^	75% ^†^	95.3% ^†^

Note: * diagnostic accuracy of endometrial sampling when compared to final histological diagnosis; ^†^ accuracy of endometrial cytology for diagnosing endometrial carcinoma and its precursors.

## Data Availability

The original data presented in the study are openly available in PubMed at https://pubmed.ncbi.nlm.nih.gov (accessed on 13 January 2025).

## References

[1] Cancer Stat Facts: Uterine Cancer. National Cancer Institute. https://seer.cancer.gov/statfacts/html/corp.html.

[2] Raglan O., Kalliala I., Markozannes G., Cividini S., Gunter M.J., Nautiyal J., Gabra H., Paraskevaidis E., Martin-Hirsch P., Tsilidis K.K. (2018). Risk factors for endometrial cancer: An umbrella review of the literature. Int. J. Cancer.

[3] Pakish J.B., Lu K.H., Sun C.C., Burzawa J.K., Greisinger A., Smith F.A., Fellman B., Urbauer D.L., Soliman P.T. (2016). Endometrial Cancer Associated Symptoms: A Case-Control Study. J. Women’s Health.

[4] Epstein E., Blomqvist L. (2014). Imaging in endometrial cancer. Best Pract. Res. Clin. Obstet. Gynaecol..

[5] Moore J.F., Carugno J. (2025). Hysteroscopy. StatPearls.

[6] McGurgan P.M., McIlwaine P. (2015). Complications of hysteroscopy and how to avoid them. Best Pract. Res. Clin. Obstet. Gynaecol..

[7] Lubbers J.A. (1977). Diagnostic Suction Curettage Without Anesthesia. Acta Obstet. Gynecol. Scand..

[8] Berek J.S., Matias-Guiu X., Creutzberg C., Fotopoulou C., Gaffney D., Kehoe S., Lindemann K., Mutch D., Concin N. (2023). FIGO staging of endometrial cancer: 2023. Int. J. Gynecol. Obstet..

[9] Abdulfatah E., Wakeling E., Sakr S., Al-Obaidy K., Bandyopadhyay S., Morris R. (2019). Molecular classification of endometrial carcinoma applied to endometrial biopsy specimens: Towards early personalized patient management. Gynecol. Oncol..

[10] Einerth Y. (1982). Vacuum curettage by the Vabrar method. A simple procedure for endometrial diagnosis. Acta Obstet. Gynecol. Scand..

[11] Kaunitz A.M., Masciello A., Ostrowski M., Rovira E.Z. (1988). Comparison of endometrial biopsy with the endometrial Pipelle and Vabra aspirator. Maturitas.

[12] Angioni S., Loddo A., Milano F., Piras B., Minerba L., Melis G.B. (2008). Detection of Benign Intracavitary Lesions in Postmenopausal Women with Abnormal Uterine Bleeding: A Prospective Comparative Study on Outpatient Hysteroscopy and Blind Biopsy. J. Minim. Invasive Gynecol..

[13] Loverro G., Bettocchi S., Cormio G., Nicolardi V., Porreca M.R., Pansini N. (1996). Diagnostic accuracy of hysteroscopy in endometrial hyperplasia. Maturitas.

[14] Zhu H., Liang X., Wang J., Cui H., Wei L. (2010). Hysteroscopy and Directed Biopsy in the Diagnosis of Endometrial Carcinoma. China Med. J..

[15] Gkrozou F., Dimakopoulos G., Vrekoussis T., Lavasidis L., Koutlas A., Navrozoglou I. (2014). Hysteroscopy in women with abnormal uterine bleeding: A meta-analysis on four major endometrial pathologies. Arch. Gynecol. Obstet..

[16] Van Kerkvoorde T.C., Veersema S., Timmermans A. (2012). Long-Term complications of office hysteroscopy: Analysis of 1028 cases. J. Minim. Invasive Gynecol..

[17] Selvaggi L., Cormio G., Ceci O., Loverro G., Cazzolla A., Bettocchi S. (2003). Hysteroscopy does not increase the risk of microscopic extrauterine spread in endometrial carcinoma. Int. J. Gynecol. Cancer.

[18] Du Y., Xu Y., Qin Z., Sun L., Chen Y., Han L. (2021). The Oncology Safety of Diagnostic Hysteroscopy in Early-Stage Endometrial Cancer: A Systematic Review and Meta-Analysis. Front. Oncol..

[19] Delke I., Veridiano N.P., Diamond B. (1980). Vabra aspiration in office gynecology. Gynecol. Oncol..

[20] Naim N.M., Mahdy Z.A., Ahmad S., Razi Z.R.M. (2007). The Vabra aspirator versus the Pipelle device for outpatient endometrial sampling. Aust. N. Z. J. Obstet. Gynaecol..

[21] Moawad N.S., Santamaria E., Johnson M., Shuster J. (2014). Cost-Effectiveness of office hysteroscopy for abnormal uterine bleeding. J. Soc. Laparosc. Robot. Surg..

[22] Breijer M.C., Van Hanegem N., Visser N.C.M., Verheijen R.H.M., Mol B.W.J., Pijnenborg J.M.A. (2015). Does probability guided hysteroscopy reduce costs in women investigated for postmenopausal bleeding?. Sci. World J..

[23] Kato M.K., Fujii E., Yamaguchi M., Higuchi D., Asami Y., Hiranuma K. (2024). Excellent concordance of the molecular classification between preoperative biopsy and final hysterectomy in endometrial carcinoma. Gynecol. Oncol..

[24] Van Den Heerik A.S.V.M., Ter Haar N.T., Vermij L., Jobsen J.J., Brinkhuis M., Roothaan S.M. (2023). QPOLE: A quick, simple, and cheap alternative for POLE sequencing in endometrial cancer by multiplex genotyping quantitative polymerase chain reaction. JCO Glob. Oncol..

[25] Di Spiezio Sardo A., Saccone G., Carugno J., Pacheco L.A., Zizolfi B., Haimovich S. (2022). Endometrial biopsy under direct hysteroscopic visualisation versus blind endometrial sampling for the diagnosis of endometrial hyperplasia and cancer: Systematic review and meta-analysis. Facts Views Vis. ObGyn.

[26] Clark T.J., Voit D., Gupta J.K., Hyde C., Song F., Khan K.S. (2002). Accuracy of hysteroscopy in the diagnosis of endometrial cancer and hyperplasia. J. Am. Med. Assoc..

[27] Sakna N.A., Elgendi M., Salama M.H., Zeinhom A., Labib S., Nabhan A.F. (2023). Diagnostic accuracy of endometrial sampling tests for detecting endometrial cancer: A systematic review and meta-analysis. BMJ Open.

[28] Dijkhuizen F.P., Mol B.W., Brolmann H.A., Heintz A.P. (2000). The accuracy of endometrial sampling in the diagnosis of patients with endometrial carcinoma and hyperplasia: A meta-analysis. Cancer.

[29] Tanko N.M., Linkov F., Bapayeva G., Ukybassova T., Kaiyrlykyzy A., Aimagambetova G. (2021). Pipelle Endometrial Biopsy for Abnormal uterine bleeding in daily Clinical practice: Why the approach to patients should be personalized?. J. Pers. Med..

[30] Elahmedawy H., Snook N.J. (2021). Complications of operative hysteroscopy: An anaesthetist’s perspective. BJA Educ..

[31] Warring S.K., Borah B., Moriarty J., Gullerud R., Lemens M.A., Destephano C. (2021). The cost of diagnosing endometrial cancer: Quantifying the healthcare cost of an abnormal uterine bleeding workup. Gynecol. Oncol..

[32] Galant N., Krawczyk P., Monist M., Obara A., Gajek Ł., Grenda A. (2024). Molecular classification of endometrial cancer and its impact on therapy selection. Int. J. Mol. Sci..

[33] Clark C., Loizzi V., Cormio G., Lopez S. (2024). Sentinel Lymph node Assessment in Endometrial Cancer: A review. Cancers.

[34] Endometrial Cancer (2015). Practice Bulletin No. 149. Obstet. Gynecol..

[35] Oaknin A., Bosse T.J., Creutzberg C.L., Giornelli G., Harter P., Joly F. (2022). Endometrial cancer: ESMO Clinical Practice Guideline for diagnosis, treatment and follow-up. Ann. Oncol..

[36] Du J., Li Y., Lv S., Wang Q., Sun C., Dong X. (2016). Endometrial sampling devices for early diagnosis of endometrial lesions. J. Cancer Res. Clin. Oncol..

